# Investigation of the Effects of Cadherin 23 and Oncomodulin on Early Progressive Hearing Loss Using a New Oncomodulin Mouse Model

**DOI:** 10.3390/ijms27093835

**Published:** 2026-04-25

**Authors:** Mi-Jung Kim, Robert J. Fuentes, Yingjie Zhou, Jing Zheng

**Affiliations:** 1 Department of Otolaryngology-Head and Neck Surgery, Feinberg School of Medicine, Northwestern University, Chicago, IL 60611, USA; mijungkim@northwestern.edu (M.-J.K.); robert.fuentes@northwestern.edu (R.J.F.); 2 Department of Communication Sciences and Disorders, School of Communication, Northwestern University, Evanston, IL 60208, USA; yingjie.zhou@northwestern.edu; 3 The Knowles Hearing Center, Northwestern University, Evanston, IL 60208, USA

**Keywords:** oncomodulin, cadherin 23, outer hair cell stress, age-related hearing loss, HPβCD

## Abstract

Oncomodulin (OCM) is the most abundant Ca^2+^ buffering protein found in mature outer hair cells (OHCs). Cadherin 23 (CDH23) is a crucial component of the tip-links in hair cell stereocilia. The absence or dysfunction of these two proteins contributes to the early onset of age-related hearing loss (AHL). In this study, we investigated the effects of the *Cdh23*^753G→A^ mutation on OHC function using new *Ocm*-knockout (KO) mouse models (*Ocm*^tm1a/tm1a^) with or without the *Cdh23*^753G→A^ mutation. Despite having the same genetic background, *Ocm*-KO mice carrying the *Cdh23*^753G→A^ mutation displayed a notable decline in OHC function across all measured frequencies as early as three months of age. In contrast, *Ocm*-KO mice without the *Cdh23*^753G→A^ mutation did not exhibit comparable hearing loss until they reached twelve months of age. Additionally, we examined the role of OCM in preserving OHC function under ototoxic stress induced by HPβCD (2-hydroxypropyl-β-cyclodextrin). The distortion product otoacoustic emission data show that the administration of HPβCD resulted in a more pronounced decline in OHC function in *Ocm*-KO mice compared to wild-type (WT) mice. Time-lapse recording also shows that HPβCD treatment led to greater structural deterioration and more rapid rupture events in OHCs from *Ocm*-KO mice than in those from WT mice. These findings suggest that the *Cdh23*^753G→A^ mutation, rather than other potential strain-specific genetic factors associated with AHL, significantly exacerbates the early onset of AHL phenotypes in *Ocm*-KO mice. Furthermore, our data indicates that the OCM protein in OHCs enhances their ability to withstand ototoxic stimuli.

## 1. Introduction

Age-related hearing loss (AHL), also known as presbycusis, is a significant global health issue that affects millions of people worldwide. Over 65% of Americans aged 71 and older suffer from hearing loss, with similar estimates worldwide [1]. In the mammalian cochlea, including humans, sound-induced vibrations of the organ of Corti (OC) open mechanoelectrical transduction (MET) channels located at the stereocilia on hair cells, allowing Ca^2+^ and other cations to enter the hair cells from the endolymph. Cadherin 23 (CDH23) is a component of the tip-links in hair cell stereocilia, interacting with protocadherin 15 to form the tip-link filaments that are thought to gate MET channels [2,3]. As true sensory receptors, inner hair cells (IHCs) transmit sound information to the brain via a neuronal network. In outer hair cells (OHCs), sound-evoked MET currents drive electromotility that amplifies the motion of the OC required for high sensitivity and sharp frequency selectivity of mammalian hearing [4,5,6,7]. The sites of age-related cochlear pathology include OHCs, IHCs, synapses, spiral ganglion neurons, and stria vascularis [8]. Human temporal bone studies indicate that AHL is primarily driven by the degeneration of inner ear sensory cells, particularly OHCs [9]. OHCs are generally the first to be damaged by common cochlear stressors, including aging, noise, and ototoxic drugs. Imbalance of intracellular Ca^2+^ homeostasis is a crucial factor contributing to the vulnerability of OHCs to cochlear insults [10]. Ca^2+^ homeostasis in OHCs is regulated by various components, including Ca^2+^-binding proteins (CaBPs). There are four major CaBPs in OHCs: parvalbumin-α, calbindin-D28k, calretinin, and oncomodulin (OCM). OCM is a small, acidic EF-hand CaBP that belongs to the parvalbumin family as the mammalian parvalbumin-β [11,12,13]. OCM is preferentially expressed in OHCs and is recognized as a dominant Ca^2+^ buffer in mature OHCs with OCM at a significantly higher concentration (2–3 mM) than other CaBPs [14,15]. The combined absence of parvalbumin-α, calbindin-D28k, and calretinin in mice has little impact on hearing [16]. However, mice without OCM display early progressive hearing loss and degeneration of OHCs, indicating that OCM is essential for maintaining cochlear function with age [17,18,19].

Inbred strains of mice vary widely in the onset and progression of AHL, making strain selection critical when assessing hearing in mutant mice [20,21,22]. A single G→A point mutation at coding nucleotide position 753 of the *Cdh23* gene (*Cdh23*^753G→A^) causes in-frame skipping of the seventh coding exon of the *Cdh23* gene. The *Cdh23* mutation contributes to early-onset AHL in common inbred mouse strains [23,24,25,26]. For example, the C57BL/6 (B6) strain has the mutant *Cdh23* genotype (*Cdh23*^753A/753A^) and exhibits high-frequency hearing loss by 3–6 months of age that progresses to profound hearing loss by 15 months. In contrast, the CBA/CaJ (CBA) strain has the wild-type (WT) *Cdh23* genotype (*Cdh23*^753G/753G^) and maintains normal hearing until 15 months of age or older [20,21,25,27]. Climer et al. have demonstrated that deletion of *Ocm* (*Ocm*^−/−^) leads to early progressive hearing loss in mice on two different genetic backgrounds: B6 and CBA. They have also observed that B6 *Ocm*^−/−^ mice experience hearing loss at an earlier age (3–4 months) than CBA *Ocm*^−/−^ mice (5–7 months) [18]. However, it is not clear whether the delayed hearing loss observed in CBA *Ocm*^−/−^ mice is solely due to the AHL-resistant *Cdh23*^753G^ allele on the CBA strain or is a result of a combination of *Cdh23*^753G^ and other strain-specific factors.

In this study, we aimed to investigate the impact of the *Cdh23*^753G→A^ mutation on cochlear function in *Ocm*-knockout (KO) mice on a uniform genetic background. We generated new *Ocm*-KO mouse models (*Ocm*^tm1a/tm1a^) with or without the *Cdh23*^753G→A^ mutation on a sighted FVB;C57BL/6N-A^tm1Brd^ (FVB;B6) genetic background. As OCM is preferentially expressed in mature OHCs in the cochlea, we measured distortion product otoacoustic emission (DPOAE) thresholds as the mice aged, alongside WT (*Ocm*^+/+^) controls. We also collected their cochleae for immunofluorescence to assess OHC loss. These histological data were compared with physiological data from the DPOAE test, which measures sounds produced by healthy, functional OHCs. To further investigate the role of OCM in maintaining cochlear function, we stressed OHCs using an ototoxic reagent called HPβCD (2-hydroxypropyl-β-cyclodextrin). HPβCD is a cyclic oligosaccharide that can sequester cholesterol. While HPβCD shows potential as a therapeutic agent for cholesterol-associated neurodegenerative diseases, including Niemann–Pick disease type C [28,29,30] and Alzheimer’s disease [28,30], it also induces ototoxicity in both humans and animal models [29,31] by causing massive OHC damage. Using both in vivo and in vitro systems, we compared the hearing and OHC response of the *Ocm*-KO mice following HPβCD treatment. Our collected data confirm that both *Cdh23*^753A^ and the absence of the OCM protein contribute to the early onset of AHL. Specifically, the *Cdh23*^753G→A^ mutation alone can further accelerate AHL in *Ocm*-KO mice. Furthermore, the lack of OCM renders OHCs more susceptible to ototoxic agents, confirming the important role of OCM in maintaining OHC function.

## 2. Results

### 2.1. Establishment of a New Ocm-KO Mouse Model: Ocm^tm1a/tm1a^

**Figure 1:**
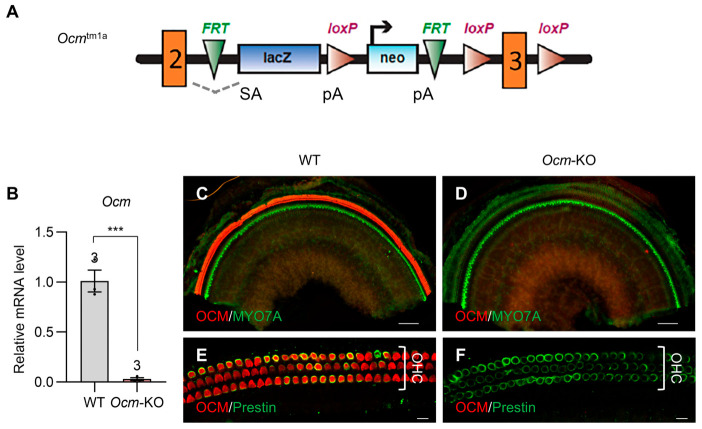
*Ocm*^tm1a^ allele leads to a highly efficient KO of the OCM protein. (**A**). Schematic diagram of the *Ocm*^tm1a^ allele structure. The *Ocm*^tm1a^ allele has RNA processing signals within a gene trapping cassette inserted into the intron between exons 2 and 3 of the *Ocm* gene. (**B**). *Ocm* mRNA level is significantly decreased in the cochlea of *Ocm*-KO mice. The levels of *Ocm* mRNA expression were measured by RT-qPCR in the cochlear tissues from WT and *Ocm*-KO mice at 2 months of age. Sample sizes: WT, *n* = 3; *Ocm*-KO, *n* = 3. Data are shown as means ± SEM. The relative mRNA level of *Ocm* was normalized to a reference gene *B2m*. Unpaired two-tailed Student’s *t*-test was performed. ***, 0.0001 ≤ *p* < 0.001. (**C**–**F**). *Ocm* protein is not expressed in the cochlea of *Ocm*-KO mice. Representative immunostaining images of the cochlear whole mounts within the frequency ranges of 19.1–36.5 kHz for (**C**,**D**) and 9.5–19.1 kHz for (**E**,**F**) from 2-month-old WT (**C**,**E**) and *Ocm*-KO (**D**,**F**) mice. Cochleae were stained for OCM (red), MYO7A (green), or prestin (green). Scale bars: 100 μm for (**C**,**D**) and 10 μm for (**E**,**F**). WT, *Cdh23*^753G/753G^;*Ocm*^+/+^ for (**B**) and *Cdh23*^753G/753A^;*Ocm*^+/+^ for (**C**,**E**), *Ocm*-KO, *Cdh23*^753G/753G^;*Ocm*^tm1a/tm1a^ for (**B**) and *Cdh23*^753G/753A^;*Ocm*^tm1a/tm1a^ for (**D**,**F**).

We generated a new *Ocm*-KO (*Ocm*^tm1a/tm1a^) mouse model based on the ‘KO-first, conditional-ready’ design [32,33,34]. In the *Ocm*^tm1a^ allele, a gene trapping cassette containing RNA processing signals was inserted into the intron between exons 2 and 3 of the *Ocm* gene (Figure 1A). It is predicted that a splice acceptor (SA) in the cassette captures the RNA transcript and a polyadenylation sequence (pA) truncates the transcript, thereby the *Ocm* gene is not transcribed into the full-length mRNA, resulting in the elimination of OCM protein synthesis. To validate this prediction, we measured the levels of *Ocm* mRNA expression by RT-qPCR in the cochleae from WT and *Ocm*-KO mice at 2 months of age. *Ocm*-KO mice showed a 97% decrease in *Ocm* mRNA expression levels in cochlear tissues compared to WT mice (Figure 1B). We also performed immunofluorescence to inspect OCM protein expression in the cochleae from 2-month-old WT and *Ocm*-KO mice. Cochlear whole mounts were stained with antibodies for OCM, a hair cell marker myosin VIIA (MYO7A), or an OHC marker prestin. As expected, OCM immunolabeling was present in the OHCs of WT mice (Figure 1C,E) but not in those of *Ocm*-KO mice (Figure 1D,F). These results indicate that the *Ocm*^tm1a^ allele leads to a highly efficient elimination of the OCM protein in the mouse cochlea, suggesting that *Ocm*^tm1a/tm1a^ is a new *Ocm*-KO mouse model that eliminates OCM protein synthesis without deleting the *Ocm* gene.

### 2.2. Ocm^tm1a/tm1a^ Mice Display Early Progressive Hearing Loss and OHC Degeneration

**Figure 2:**
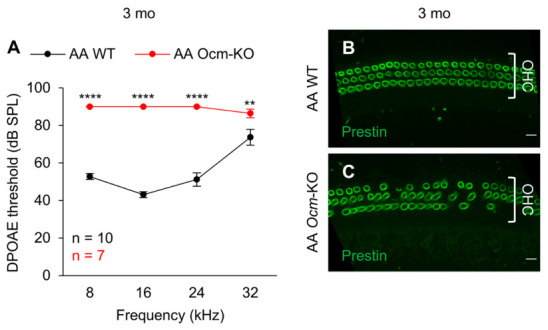
*Ocm*^tm1a/tm1a^ mice carrying a *Cdh23*^753G→A^ mutation display profound hearing loss and loss of OHCs at 3 months. (**A**). The function of OHCs in AA *Ocm*-KO mice is significantly compromised at 3 months. DPOAE thresholds were measured at 8, 16, 24, and 32 kHz in AA WT and AA *Ocm*-KO mice at 3 months of age. Sample sizes: AA WT, *n* = 10; AA *Ocm*-KO, *n* = 7. Data are shown as means ± SEM. Two-way ANOVA with Bonferroni’s multiple comparisons test was performed. **, 0.001 ≤ *p* < 0.01, ****, *p* < 0.0001. (**B**,**C**). AA *Ocm*-KO mice lose OHCs at 3 months. Representative immunostaining images of the cochlear whole mounts show OHC loss in AA *Ocm*-KO mice (**C**) compared to OHCs from AA WT mice (**B**). The samples were collected within the frequency range of 19.1–36.5 kHz from 3-month-old mice. Cochleae were stained for prestin. Scale bar: 10 μm. mo, month, AA WT, *Cdh23*^753A/753A^;*Ocm*^+/+^, AA *Ocm*-KO, *Cdh23*^753A/753A^;*Ocm*^tm1a/tm1a^.

The B6 strain has the mutant *Cdh23* genotype (*Cdh23*^753A/753A^) and exhibits high-frequency hearing loss by 3–6 months of age that progresses to profound hearing loss by 15 months [20,21,25,27]. In contrast, the FVB strain has the WT *Cdh23* genotype (*Cdh23*^753G/753G^) and displays good hearing at 7 months [21,25]. The newly established *Ocm*-KO mice were on an FVB;B6 genetic background with or without a single G→A point mutation at coding nucleotide position 753 of the *Cdh23* gene (*Cdh23*^753G→A^). To assess the OHC function of the *Ocm*-KO mice carrying the *Cdh23*^753G→A^ mutation, we measured DPOAE thresholds at 8, 16, 24, and 32 kHz in AA WT (*Cdh23*^753A/753A^;*Ocm*^+/+^) and AA *Ocm*-KO (*Cdh23*^753A/753A^;*Ocm*^tm1a/tm1a^) mice at 1 and 3 months of age. At 1 month of age, there were no differences in DPOAE thresholds at 8–32 kHz between AA WT and AA *Ocm*-KO mice. However, at 3 months of age, DPOAE thresholds for AA *Ocm*-KO mice were at or near 90 dB SPL and 13–47 dB higher than those for AA WT mice at 8–32 kHz (Figure 2A), indicating that AA *Ocm*-KO mice display profound hearing loss at 3 months. We note that AA WT mice also had high-frequency hearing loss at 3 months, as often observed in B6 mice [20,21,27]. We then performed immunofluorescence to inspect OHC loss in the cochleae from 3-month-old AA WT and AA *Ocm*-KO mice. Cochlear whole mounts within the frequency range of 19.1–36.5 kHz were stained with anti-prestin. As shown in Figure 2B,C, 3-month-old AA *Ocm*-KO mice showed more OHC loss compared to age-matched AA WT mice. This data is consistent with the DPOAE hearing test results. These findings are largely consistent with previously published data collected from *Ocm*-KO mouse models on the B6 background [17,19], which showed significantly higher DPOAE thresholds at 12–30 kHz than WT mice at 8 weeks [19] and DPOAE thresholds at or near the measurement ceiling for 5.6–45.2 kHz at 14–26 weeks [17].

**Figure 3:**
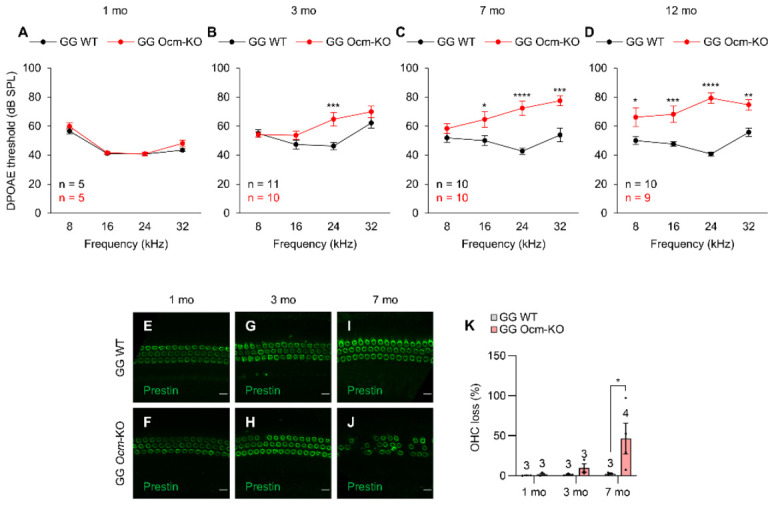
*Ocm*^tm1a/tm1a^ mice lacking a *Cdh23*^753G→A^ mutation show a delay in profound hearing loss and loss of OHCs. (**A**–**D**). GG *Ocm*-KO mice display high frequency hearing loss at 3 months that progresses to low- to high-frequency hearing loss by 12 months. DPOAE thresholds were measured at 8, 16, 24, and 32 kHz in GG WT and GG *Ocm*-KO mice at 1 (**A**), 3 (**B**), 7 (**C**), and 12 (**D**) months of age. Sample sizes: at 1 mo: GG WT, *n* = 5, GG *Ocm*-KO, *n* = 5; at 3 mo: GG WT, *n* = 11, GG *Ocm*-KO, *n* = 10; at 7 mo: GG WT, *n* = 10, GG *Ocm*-KO, *n* = 10; at 12 mo: GG WT, *n* = 10, GG *Ocm*-KO, *n* = 9. (**E**–**K**). GG *Ocm*-KO mice lose OHCs at 7 months. Representative immunostaining images of the cochlear whole mounts within the frequency range of 19.1–36.5 kHz from 1-, 3-, and 7-month-old GG WT (**E**,**G**,**I**) and GG *Ocm*-KO (**F**,**H**,**J**) mice. Cochleae were stained for prestin. Scale bar: 10 μm. (**K**). A histogram showing average OHC loss observed in immunostaining samples ranging from 1 to 7 months. Sample sizes: at 1 mo: GG WT, *n* = 3, GG *Ocm*-KO, *n* = 3; at 3 mo: GG WT, *n* = 3, GG *Ocm*-KO, *n* = 3; at 7 mo: GG WT, *n* = 3, GG *Ocm*-KO, *n* = 4. Data are shown as means ± SEM. Two-way ANOVA with Bonferroni’s multiple comparisons tests were performed. *, 0.01 ≤ *p* < 0.05, **, 0.001 ≤ *p* < 0.01, ***, 0.0001 ≤ *p* < 0.001, ****, *p* < 0.0001. mo, month, GG WT, *Cdh23*^753G/753G^;*Ocm*^+/+^, GG *Ocm*-KO, *Cdh23*^753G/753G^;*Ocm*^tm1a/tm1a^.

To assess the OHC function of the *Ocm*-KO mice lacking the *Cdh23*^753G→A^ mutation, we also measured DPOAE thresholds at 8, 16, 24, and 32 kHz in GG WT (*Cdh23*^753G/753G^;*Ocm*^+/+^) and GG *Ocm*-KO (*Cdh23*^753G/753G^;*Ocm*^tm1a/tm1a^) mice at 1, 3, 7, and 12 months of age. At 1 month of age, there were no differences in DPOAE thresholds at 8–32 kHz between GG WT and GG *Ocm*-KO mice (Figure 3A), similar to the case of AA WT and AA *Ocm*-KO mice. However, at 3 months of age, GG *Ocm*-KO mice showed an 18 dB increase in DPOAE thresholds at 24 kHz compared to GG WT mice (Figure 3B), indicating that GG *Ocm*-KO mice display high-frequency hearing loss at 3 months due to the lack of OCM in OHCs. Furthermore, at 7 months of age, DPOAE thresholds for GG *Ocm*-KO mice were 15–30 dB higher than those for GG WT mice at 16–32 kHz (Figure 3C), indicating that GG *Ocm*-KO mice display middle- to high- frequency hearing loss at 7 months. At 12 months of age, GG *Ocm*-KO mice also showed a 16–39 dB increase in DPOAE thresholds at 8–32 kHz compared to GG WT mice (Figure 3D), indicating that GG *Ocm*-KO mice display hearing loss affecting all frequencies at 12 months. Consistently, auditory brainstem response (ABR) data show that GG *Ocm*-KO mice have normal hearing at 1 month but display low- to high-frequency hearing loss at 12 months (Supplementary Figure S1A,B).

We also performed immunofluorescence to inspect and quantify OHC loss in the cochleae from 1-, 3-, and 7-month-old GG WT and GG *Ocm*-KO mice. Cochlear whole mounts within the frequency range of 19.1–36.5 kHz were stained with anti-prestin. GG WT mice maintained stable OHC numbers with only 0–2% OHC loss from 1 to 7 months of age (Figure 3E,G,I,K). In contrast, GG *Ocm*-KO mice showed 2%, 10%, and 47% OHC loss at 1, 3, and 7 months of age, respectively (Figure 3F,H,J,K). At 1 and 3 months of age, there were no significant differences in OHC loss between GG WT and GG *Ocm*-KO mice (Figure 3E–H,K). However, at 7 months of age, GG *Ocm*-KO mice showed significantly more OHC loss compared to age-matched GG WT mice (Figure 3I–K). Taken together, these results indicate that *Ocm*-KO mice have normal hearing at 1 month but display early progressive hearing loss and OHC degeneration regardless of the presence or absence of a *Cdh23*^753G→A^ mutation. However, *Ocm*-KO mice without the *Cdh23* mutation display delayed hearing loss and OHC loss compared to *Ocm*-KO mice with this mutation.

### 2.3. Cdh23^753A^ Alone Further Accelerates Age-Related Hearing Loss in Ocm^tm1a/tm1a^ Mice

**Figure 4:**
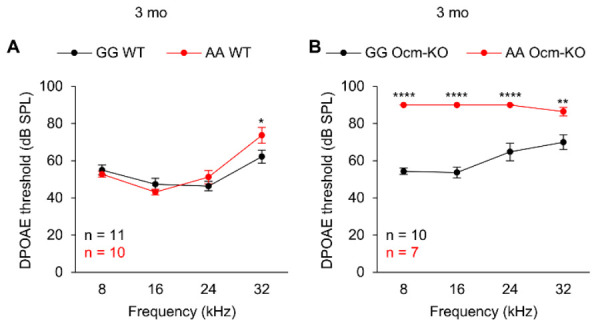
A *Cdh23*^753G→A^ mutation leads to a greater decline in OHC function in *Ocm*^tm1a/tm1a^ mice than in *Ocm*^+/+^ mice at 3 months. (**A**,**B**). DPOAE thresholds were measured at 8, 16, 24, and 32 kHz in GG WT and AA WT (**A**) and GG *Ocm*-KO and AA *Ocm*-KO (**B**) mice at 3 months of age. Sample sizes: GG WT, *n* = 11; AA WT, *n* = 10; GG *Ocm*-KO, *n* = 10; AA *Ocm*-KO, *n* = 7. Data are shown as means ± SEM. Two-way ANOVA with Bonferroni’s multiple comparisons tests was performed. *, 0.01 ≤ *p* < 0.05, **, 0.001 ≤ *p* < 0.01, ****, *p* < 0.0001. mo, month, GG WT, *Cdh23*^753G/753G^;*Ocm*^+/+^, AA WT, *Cdh23*^753A/753A^;*Ocm*^+/+^, GG *Ocm*-KO, *Cdh23*^753G/753G^;*Ocm*^tm1a/tm1a^, AA *Ocm*-KO, *Cdh23*^753A/753A^;*Ocm*^tm1a/tm1a^.

Our data confirm that OCM is not required for the development of normal hearing, as previously reported [17,18,19]. Despite both types of mice having the same genomic background, *Ocm*-KO mice carrying the *Cdh23*^753G→A^ mutation experience profound hearing loss at 3 months (Figure 2A), whereas *Ocm*-KO mice without this mutation do not exhibit comparable hearing loss until 12 months (Figure 3D). As shown in Figure 4A, 3-month-old AA WT mice showed a significant increase (11 dB) in DPOAE thresholds at 32 kHz compared to age-matched GG WT mice, indicating that a *Cdh23*^753G→A^ mutation alone leads to a decline in high-frequency OHC function in WT mice as early as 3 months. The impact of the *Cdh23*^753G→A^ mutation on OHC function is even more pronounced when OHCs lack OCM. As shown in Figure 4B, 3-month-old AA *Ocm*-KO mice exhibited a 16–36 dB increase in DPOAE thresholds at all measured frequencies compared to age-matched GG *Ocm*-KO mice, indicating that a *Cdh23*^753G→A^ mutation leads to a significant decline in low- to high-frequency OHC function in *Ocm*-KO mice at 3 months. Taken together, these results suggest that *Cdh23*^753A^ can further accelerate the early onset of AHL in *Ocm*-KO mice.

### 2.4. Ocm^tm1a/tm1a^ Mice Are More Susceptible to HPβCD-Induced Hearing Loss

**Figure 5:**
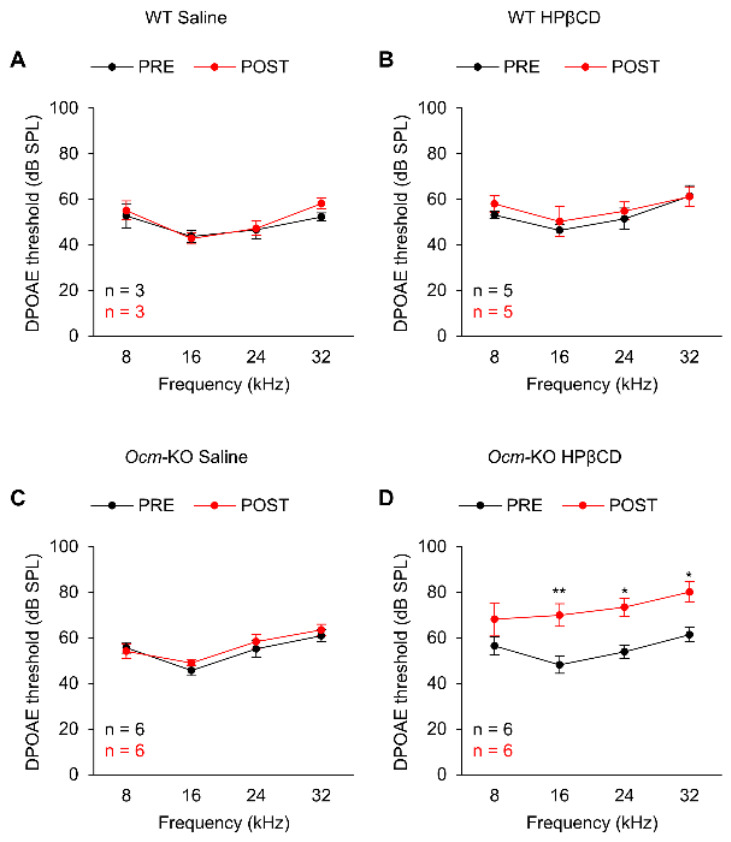
HPβCD treatment results in greater hearing loss in *Ocm*^tm1a/tm1a^ mice. (**A**–**D**). DPOAE thresholds were measured at 8, 16, 24, and 32 kHz in 2-month-old WT (**A**,**B**) and *Ocm*-KO (**C**,**D**) mice 7 days before (PRE) and 4 h after (POST) either saline (**A**,**C**) or 8000 mg/kg HPβCD (**B**,**D**) single subcutaneous injection. Sample sizes: WT Saline, *n* = 3; WT HPβCD, *n* = 5; *Ocm*-KO Saline, *n* = 6; *Ocm*-KO HPβCD, *n* = 6. Data are shown as means ± SEM. Two-way ANOVA with Bonferroni’s multiple comparisons tests was performed. *, 0.01 ≤ *p* < 0.05, **, 0.001 ≤ *p* < 0.01. WT, *Cdh23*^753G/753A^;*Ocm*^+/+^, *Ocm*-KO, *Cdh23*^753G/753A^;*Ocm*^tm1a/tm1a^.

A body of evidence shows that HPβCD causes rapid hearing loss and massive OHC damage in a dose-dependent manner in mice [35,36,37,38,39,40] and rats [41,42,43,44,45]. We and other research groups have reported that mice injected with a single subcutaneous dose of 8000 mg/kg HPβCD exhibit significant hearing loss within 1 week [35,37,38,39]. Our group has also observed significant OHC loss in mice as early as 24 h after 8000 mg/kg HPβCD single subcutaneous injection [40]. However, no significant OHC loss is observed in mice 4 h after 8000 mg/kg HPβCD single subcutaneous injection, but the OHCs undergo structural deterioration, showing uneven prestin staining [40]. To investigate whether the absence of OCM in mice affects OHC’s ability to resist ototoxic stress, we measured DPOAE thresholds at 8, 16, 24, and 32 kHz in 2-month-old WT and *Ocm*-KO mice 7 days before and 4 h after either saline or 8000 mg/kg HPβCD single subcutaneous injection. We used WT and *Ocm*-KO mice heterozygous for the *Cdh23*^753G→A^ mutation. We confirmed that there were no differences in DPOAE thresholds at 8–32 kHz between WT and *Ocm*-KO mice heterozygous for the *Cdh23*^753G→A^ mutation at 2 months of age. WT mice did not show changes in DPOAE thresholds after either saline (Figure 5A) or HPβCD (Figure 5B) injection. However, *Ocm*-KO mice showed a 19–22 dB increase in DPOAE thresholds at 16–32 kHz after HPβCD injection (Figure 5D), while saline injection did not change DPOAE thresholds in *Ocm*-KO mice (Figure 5C), indicating that HPβCD treatment leads to more hearing loss in *Ocm*-KO mice compared to WT mice. These data suggest that *Ocm*-KO mice are more susceptible to HPβCD-induced hearing loss compared to WT mice.

### 2.5. Ocm^tm1a/tm1a^ OHCs Are More Susceptible to HPβCD-Induced Structural Deterioration In Vivo

**Figure 6:**
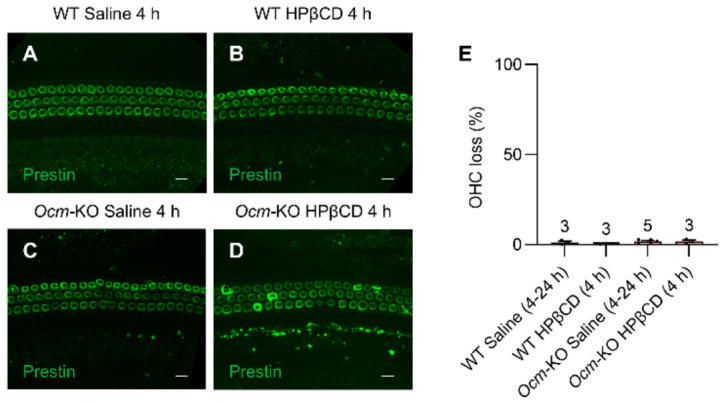
*Ocm*^tm1a/tm1a^ OHCs undergo more structural deterioration under HPβCD treatment in vivo. (**A**–**D**). Representative immunostaining images of the cochlear whole mounts within the frequency range of 19.1–36.5 kHz from 2-month-old WT (**A**,**B**) and *Ocm*-KO (**C**,**D**) mice 4 h after either saline (**A**,**C**) or 8000 mg/kg HPβCD (**B**,**D**) single subcutaneous injection. Cochleae were stained for prestin. Scale bar: 10 μm. (**E**). A histogram showing average OHC loss observed in immunostaining samples treated with HPβCD or saline at different time points. Sample sizes: WT Saline (4–24 h), *n* = 3; WT HPβCD (4 h), *n* = 3; *Ocm*-KO Saline (4–24 h), *n* = 5; *Ocm*-KO HPβCD (4 h), *n* = 3. Data are shown as means ± SEM. One-way ANOVA with Tukey’s multiple comparisons test was performed. h, hour, WT, *Cdh23*^753G/753A^;*Ocm*^+/+^, *Ocm*-KO, *Cdh23*^753G/753A^;*Ocm*^tm1a/tm1a^.

To investigate whether the absence of OCM in mice affects OHC viability and morphology under HPβCD treatment conditions in vivo, we performed immunofluorescence in the cochleae from WT and *Ocm*-KO mice 4 or 24 h after either saline or 8000 mg/kg HPβCD single subcutaneous injection. Cochlear whole mounts within the frequency range of 19.1–36.5 kHz were stained with anti-prestin. As expected, there was minimal OHC loss in WT and *Ocm*-KO mice injected with saline regardless of the time when the cochleae were collected (Figure 6A,C,E). Significant OHC loss was observed in both WT and *Ocm*-KO mice 24 h after HPβCD injection. No significant OHC loss was also observed in WT or *Ocm*-KO mice 4 h after HPβCD injection (Figure 6B,D,E). As shown in Figure 6A,B, OHCs from WT mice showed smooth prestin staining, indicating that the OHCs are structurally intact. In contrast, OHCs from HPβCD-injected *Ocm*-KO mice showed more uneven prestin staining and variations in cell diameter compared to those from HPβCD-injected WT mice (Figure 6B,D), indicating that *Ocm*-KO OHCs undergo more structural deterioration compared to WT OHCs under HPβCD treatment conditions in vivo. In other words, while OHCs from HPβCD-injected *Ocm*-KO mice are still present in the OC, their function is compromised, as indicated by the DPOAE data. These results imply that *Ocm*-KO OHCs are more susceptible to HPβCD-induced structural deterioration compared to WT OHCs in vivo.

### 2.6. Ocm^tm1a/tm1a^ OHCs Are More Susceptible to HPβCD-Induced Rupture In Vitro

**Figure 7:**
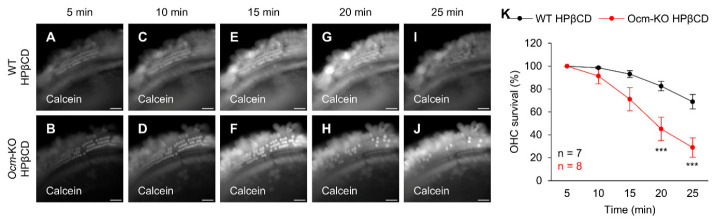
*Ocm*^tm1a/tm1a^ OHCs undergo faster rupture events under HPβCD treatment in vitro. (**A**–**J**). Representative time-lapse images of the isolated OC within the apical region of the cochlea from 1-month-old WT (**A**,**C**,**E**,**G**,**I**) and *Ocm*-KO (**B**,**D**,**F**,**H**,**J**) mice in the presence of 1 mM HPβCD. The isolated OC was labeled with calcein. The OC images captured 5 ((**A**,**B**), initial images), 10 (**C**,**D**), 15 (**E**,**F**), 20 (**G**,**H**), and 25 (**I**,**J**) minutes after exposure to HPβCD are shown. Scale bar: 20 μm. (**K**). OHC survival curve under HPβCD treatment in vitro. OHCs were counted in the isolated OC within the apical region of the cochlea from 1-month-old WT and *Ocm*-KO mice 5, 10, 15, 20, and 25 min after exposure to 1 mM HPβCD. Sample sizes: WT HPβCD, *n* = 7; *Ocm*-KO HPβCD, *n* = 8. Data are shown as means ± SEM. Two-way ANOVA with Bonferroni’s multiple comparisons test was performed. ***, 0.0001 ≤ *p* < 0.001. min, minute, WT, *Cdh23*^753G/753G^;*Ocm*^tm1c/tm1c^ for WT HPβCD (*n* = 3) in (**K**) and *Cdh23*^753G/753G^;*Ocm*^+/+^ for the others, *Ocm*-KO, *Cdh23*^753G/753G^;*Ocm*^tm1a/tm1a^.

To verify whether OHCs from *Ocm*-KO mice are more vulnerable to ototoxic stimulation, we performed time-lapse recording in the isolated OC from 1-month-old WT and *Ocm*-KO mice in the presence of 1 mM HPβCD. Isolated OC within the apical region of the cochlea were labeled using a cell-permeant vital dye calcein AM (acetoxymethyl ester) to identify viable OHCs with intact plasma membranes under in vitro HPβCD treatment conditions. Calcein AM is non-fluorescent until it enters live cells with intact plasma membranes, where intracellular esterases cleave it into green-fluorescent calcein, which is then cell-impermeable and trapped in the cytoplasm [46]. Calcein AM has been used to assess the viability of cochlear hair cells [47,48] and to evaluate the cytotoxicity of HPβCD on cell lines [49,50,51]. WT and *Ocm*-KO mice without the *Cdh23*^753G→A^ mutation were used for the experiment. Time-lapse image recording of the OC was initiated 5 min after exposure to HPβCD. The OC images were captured with a 1 s interval for 20 min. “OHC swelling followed by rupture” events were observed in the isolated OC from both WT (Supplementary Movie S1) and *Ocm*-KO (Supplementary Movie S2) mice in the presence of 1 mM HPβCD. We also counted surviving OHCs from both WT and *Ocm*-KO samples at 5, 10, 15, 20, and 25 min time points after exposure to 1 mM HPβCD. The percentage of the OHC survival was calculated based on the OHC number in initial OC image for each sample (Figure 7A,B). OC isolated from WT mice showed OHC survival of 99%, 93%, 83%, and 69% at 10, 15, 20, and 25 min after exposure to HPβCD, respectively (Figure 7C,E,G,I,K). In contrast, OC isolated from *Ocm*-KO mice exhibited OHC survival of 91%, 71%, 45%, and 29% at the same time points (Figure 7D,F,H,J,K). *Ocm*-KO samples showed a statistically significant reduction in OHC survival compared to WT controls 20 and 25 min after exposure to HPβCD (Figure 7G–K). Together, these data indicate that OHCs from *Ocm*-KO mice undergo faster rupture events compared to OHCs from WT mice during in vitro HPβCD treatment. These results align with in vivo sample data (Figure 6), showing that *Ocm*-KO OHCs are more prone to HPβCD-induced rupture compared to WT OHCs in vitro.

## 3. Discussion

In this study, we developed a new *Ocm*-KO mouse model (*Ocm*^tm1a/tm1a^) using the ‘KO-first, conditional-ready’ strategy (Figure 1A) [32,33,34]. We validated this new *Ocm*-KO model at mRNA (Figure 1B), protein (Figure 1C–F), and physiological (Figure 2, Figure 3 and Figure 4) levels. Our data indicate that OCM is not necessary for establishing hearing function but is essential for maintaining normal, healthy OHCs. This finding aligns with conclusions from targeted gene deletion *Ocm*-KO mouse models [17,18]. More importantly, our *Ocm*^tm1a^ allele contains a *loxP*-flanked critical exon 3 of the *Ocm* gene as well as an *FRT*-flanked gene trapping cassette, making it a versatile tool for generating tissue- or time-specific *Ocm*-conditional KO mice. This is particularly important to further investigate the function of OCM. Research has demonstrated that OCM can be expressed and secreted by activated macrophages and neutrophils in response to injuries in the eye [52,53,54] and spinal cord [55,56]. OCM has been recognized as a neurotrophic factor in various nervous tissues, including the retina and spinal cord. Conducting conditional KO studies of OCM would be vital for determining whether OCM secreted by different cell types could stimulate the regrowth of injured cochlear spiral ganglion neurons in the future.

CDH23 is a non-classical cadherin that is a crucial component of the tip-links in the stereocilia of inner ear hair cells, where it plays an essential role in gating MET channels [2,3]. In humans, *CDH23* gene mutations cause Usher syndrome type 1D and nonsyndromic deafness DFNB12 [57,58]. Both mouse models and human data underscore the critical role of CDH23 for hearing. In a previous study, *Ocm*-KO mice on the B6 strain experienced hearing loss at an earlier age (3–4 months) compared to *Ocm*-KO mice on the CBA strain (5–7 months) [18]. It is unclear whether the difference in AHL progression between CBA *Ocm*-KO mice (slower) and B6 *Ocm*-KO mice (more rapid) is solely due to a *Cdh23*^753G→A^ mutation or is combined with other possible strain-specific genetic contributors to AHL. In this study, we tested the impact of *Cdh23*^753G→A^ on OHC function in *Ocm*-KO mice derived from the same genetic background. Our data (Figure 2, Figure 3 and Figure 4) suggest that a *Cdh23*^753G→A^ mutation is a major contributor to exacerbating the AHL phenotypes of *Ocm*-KO mice. Our findings are in line with previous reports that *Cdh23*^753A>G^ single-nucleotide substitutions attenuate AHL in B6 mice [59,60,61,62], in the sense that the presence or absence of a *Cdh23*^753G→A^ mutation makes a significant difference in hearing in mice on a uniform genetic background.

Dysregulated intracellular Ca^2+^ homeostasis is implicated in hair cell death and hearing loss induced by ototoxic drugs, including aminoglycosides [63,64,65] and cisplatin [66,67,68]. However, there is limited knowledge regarding the role of Ca^2+^ buffering proteins, particularly OCM, in the vulnerability of OHCs to ototoxic agents. Our data from both in vitro and in vivo experiments indicate that the absence of OCM increases the susceptibility of OHCs to the ototoxic agent HPβCD. This response is similar to that observed in OHCs from *Ocm*-KO mice following noise exposure [69]. As OCM is the most abundant CaBP in OHCs, its absence leads to increased expression of multiple CaBPs and Ca^2+^-regulating purinergic receptors, including parvalbumin-α, sorcin, P2RX2, P2RX3, and P2RX7, to compensate for the loss of OCM’s function [70,71]. However, these compensatory proteins may not effectively manage excess Ca^2+^ under stressful conditions, such as exposure to HPβCD or excess noise exposure. The disruption of intracellular Ca^2+^ homeostasis is likely to result in OHCs from *Ocm*-KO mice being more susceptible to stress treatment. Nevertheless, this hypothesis needs further verification.

In summary, we have developed a new *Ocm* transgenic mouse model to further investigate the multiple functions of OCM. Our data confirm that without OCM, OHCs become more susceptible to stress conditions, resulting in the early onset of AHL. Additionally, our data suggest that a *Cdh23*^753G→A^ mutation is a major contributor that exacerbates the earlier onset of AHL in *Ocm*-KO mice.

## 4. Materials and Methods

### 4.1. Animals

All experimental procedures were conducted in accordance with the Guide for the Care and Use of Laboratory Animals by NIH and approved by Northwestern University’s Institutional Animal Care and Use Committee. To generate a new *Ocm*-KO mouse model based on the ‘KO-first, conditional-ready’ design [32,33,34], *Ocm*^tm1a(EUCOMM)Wtsi^ (*Ocm*^tm1a^, MGI: 4431716), embryonic stem cells (C57BL/6N-A^tm1Brd^-derived JM8A3.N1) that target the *Ocm* gene were purchased from the Wellcome Sanger Institute (Cambridgeshire, UK) and injected into the blastocysts of C57BL/6 mice by the Transgenic and Targeted Mutagenesis Laboratory at Northwestern University. A chimeric male mouse was mated with sighted FVB female mice (JAX: 004828) to produce mice on an FVB;B6 genetic background. FVB mice have the WT *Cdh23* genotype (*Cdh23*^753G/753G^) while B6 mice have the mutant *Cdh23* genotype (*Cdh23*^753A/753A^) [25]. To establish the *Ocm*^tm1a^ strain with or without the *Cdh23*^753G→A^ mutation, F1 mice heterozygous for both *Cdh23* and *Ocm* (*Cdh23*^753G/753A^;*Ocm*^tm1a/+^) were used for brother x sister matings to produce F2 mice. F3 to F5 mice were produced by heterozygous or homozygous cross. Most data were collected from the following mice: *Cdh23*^753A/753A^;*Ocm*^+/+^ (AA WT), *Cdh23*^753A/753A^;*Ocm*^tm1a/tm1a^ (AA *Ocm*-KO), *Cdh23*^753G/753G^;*Ocm*^+/+^ (GG WT), *Cdh23*^753G/753G^;*Ocm*^tm1a/tm1a^ (GG *Ocm*-KO), *Cdh23*^753G/753A^;*Ocm*^+/+^ (GA WT), and *Cdh23*^753G/753A^;*Ocm*^tm1a/tm1a^ (GA *Ocm*-KO). The *Ocm*^tm1a^ strain was further mated with ACTB:FLPe B6J mice (JAX: 005703) to generate the *Ocm*^tm1c^ strain that restores *Ocm* gene expression by the FLP-FRT recombination (Supplementary Figure S2A). We confirmed that *Ocm*^tm1c/tm1c^ mice expressed OCM protein in the OHCs of the cochlea (Supplementary Figure S2B,C) and displayed normal hearing at 12 months. Data collected from 3 *Cdh23*^753G/753G^;*Ocm*^tm1c/tm1c^ mice were added to Figure 7K. Genotyping was outsourced to Transnetyx. Both males and females were used in this study.

### 4.2. RT-qPCR

Mice were euthanized with CO_2_ and cochleae were dissected out. Total RNA was isolated from cochlear tissues using the Quick-RNA Miniprep Plus Kit (Zymo Research, Irvine, CA, USA), and cDNA was synthesized using the SuperScript IV VILO Master Mix (Thermo Fisher Scientific, Waltham, MA, USA) according to the manufacturer’s instructions. 100 ng of total RNA was used for cDNA synthesis in a 20 μL reaction. qPCR was performed using the PowerTrack SYBR Green Master Mix (Thermo Fisher Scientific) on the QuantStudio 7 Flex Real-Time PCR System (Thermo Fisher Scientific) according to the manufacturer’s instructions. The relative mRNA level of *Ocm* was normalized to a reference gene, *B2m* (beta-2 microglobulin) [72], and calculated using the 2^−ΔΔCt^ method [73]. Primer sequences for qPCR were as follows: *Ocm* forward 5′-ATGAGCATCACGGACATTCTGAGC-3′, *Ocm* reverse 5′-CTGGCAGACATCTTGGAGAGGC-3′, *B2m* forward 5′-TGGTCTTTCTGGTGCTTGTC-3′, and *B2m* reverse 5′-GGGTGGAACTGTGTTACGTAG-3′ [71].

### 4.3. Immunofluorescence

Mice were euthanized with Euthasol (200 mg/kg). Cochleae were dissected out and fixed with 4% formaldehyde in PBS for 2 h at room temperature or overnight at 4 °C. Cochleae were then decalcified with 10% EDTA (pH 7.4) for 2 days at 4 °C and micro-dissected into 5 pieces for whole mount processing as we previously described [40,74]. Cochlear whole mounts were blocked with 5% normal donkey serum, 0.2% saponin in TBS (tris-buffered saline) for 1 h at room temperature and incubated overnight at 4 °C with the following primary antibodies diluted in 1% normal donkey serum, 0.2% saponin in TBS: goat anti-OCM (Santa Cruz Biotechnology, Dallas, TX, USA, sc-7446, 1:500), rabbit anti-MYO7A (myosin VIIA, Proteus BioSciences, Ramona, CA, USA, 25-6790, 1:200), or rabbit anti-prestin (N-terminus of mouse prestin [75], 1:1000). Cochlear samples were then incubated for 2 h at room temperature with the following secondary antibodies diluted in 1% normal donkey serum, 0.2% saponin in TBS: donkey anti-goat Alexa Fluor 546 (Thermo Fisher Scientific, A-11056, 1:500) or donkey anti-rabbit Alexa Fluor 488 (Thermo Fisher Scientific, A-21206, 1:500) and mounted with the Dako Fluorescence Mounting Medium (Agilent Technologies, Santa Clara, CA, USA). Images were captured using a fluorescence microscope (Keyence, Osaka, Japan, BZ-X810) or a confocal microscope (Nikon, Tokyo, Japan, ECLIPSE Ti) with 20X or 60X objectives controlled by the BZ-X800 Viewer (Keyence) or the NIS-Elements (Nikon), respectively. OHC counting was performed as we previously described [40,74]. The OHCs stained with anti-prestin or anti-MYO7A were counted using ImageJ 2.14.0/1.54f (NIH) in the cochlear segment 3 that corresponds to the frequency range of 19.1–36.5 kHz. The percentage of OHC loss was then calculated based on the average OHC number in the cochlear segment 3 collected from 5 sighted FVB mice.

### 4.4. DPOAE

Distortion product otoacoustic emission (DPOAE) tests were performed using the RZ6 Multi I/O Processor (Tucker-Davis Technologies, TDT, Alachua, FL, USA) controlled by the BioSigRZ (TDT) according to the manufacturer’s instructions. Mice were anesthetized with ketamine (100 mg/kg) and xylazine (10 mg/kg) by intraperitoneal injection. During testing, body temperature was maintained using a heating pad. The ear tip of the ER10B+ microphone system (Etymotic Research, Elk Grove Village, IL, USA) that was connected to two MF1 speakers (TDT) was positioned in the left ear canal. The level of the distortion product at 2f1-f2 was collected by presenting two primary tones. The tone frequencies f1 and f2 had a f2/f1 ratio of 1.2 and were geometrically centered about 8, 16, 24, and 32 kHz. At each center frequency, the tone levels L1 and L2 remained equal and were reduced in 10 dB steps from 80 to 20 dB SPL. To analyze DPOAE data, a Fast Fourier Transform (FFT) was performed using a modified version of the MATLAB R2023a (MathWorks) code developed by Tan et al. [76]. The DPOAE input-output function was acquired, and the DPOAE threshold was defined as the level of f1 and f2 (L1 = L2) required to produce a DPOAE of 0 dB SPL [38]. If no DPOAE response was detected at the maximum stimulus level, the threshold was arbitrarily assigned as a value one step size greater than the maximum stimulus level [77].

### 4.5. ABR

Auditory brainstem response (ABR) tests were performed using the RZ6 Multi I/O Processor (TDT) controlled by the BioSigRZ (TDT) according to the manufacturer’s instructions. Mice were anesthetized with ketamine (100 mg/kg) and xylazine (10 mg/kg) by intraperitoneal injection. During testing, body temperature was maintained using a heating pad. Subdermal needle electrodes, connected to the Medusa4Z amplifier (TDT), were placed at the vertex (reference), ipsilateral ear (channel 1), and hind hip (ground). A single MF1 speaker (TDT) was positioned in the left ear canal. ABR waveforms were collected in response to tone stimuli at 8, 16, 24, 32, and 40 kHz. At each frequency, the sound level was reduced in 5 dB steps from 85 to 20 dB SPL. The ABR threshold was defined as the lowest stimulus level that produced a visually identifiable waveform. If no ABR response was detected at the maximum stimulus level, the threshold was arbitrarily assigned as a value one step size greater than the maximum stimulus level [77].

### 4.6. HPβCD Treatment In Vivo

HPβCD (2-hydroxypropyl-β-cyclodextrin, MilliporeSigma, Burlington, MA, USA, H107) was dissolved in saline (0.9% Sodium Chloride Injection, USP, ICU Medical, San Clemente, CA, USA). Both HPβCD and vehicle control solutions were filter sterilized. Mice were injected with a single subcutaneous dose of either 8000 mg/kg (high dose) HPβCD or equivalent vehicle control as we previously described [37,38,40]. A major adverse effect of using high doses of HPβCD to treat Niemann–Pick disease type C is hearing loss [29,31]. DPOAE tests were performed 7 days before and 4 h after injection. Cochleae were collected 4 or 24 h after injection for immunofluorescence.

### 4.7. HPβCD Treatment In Vitro

In vitro analysis of HPβCD-treated WT and *Ocm*-KO OHCs was performed by time-lapse recording, as we previously described [37]. A cell-permeant vital dye calcein AM (acetoxymethyl este) (Thermo Fisher Scientific, C3100), was used to identify viable OHCs with intact plasma membranes under in vitro HPβCD treatment conditions. HPβCD (MilliporeSigma, H107) was dissolved in HBSS (Hanks’ Balanced Salt Solution, Thermo Fisher Scientific, 14025). Calcein AM (Thermo Fisher Scientific, C3100) was dissolved in DMSO (Thermo Fisher Scientific, D12345). Mice were euthanized with CO_2_ and cochleae were dissected out. The OC within the apical region of the cochlea were carefully removed as we previously described [78]. The isolated OC was placed in HBSS containing 1 mM HPβCD and 1 μM calcein AM/0.1% DMSO. The osmolality of the solution was measured to ensure that it is 310 mmol/kg. Imaging of OC was initiated 5 min after exposure to HPβCD. The OC images were captured with a 1 s interval for 20 min using an inverted fluorescence microscope (Leica, Wetzlar, Germany, DM IRB) with a 40X objective controlled by the Micro-Manager [79]. The OHCs labeled with calcein were counted using ImageJ (NIH) in the OC images captured 5, 10, 15, 20, and 25 min after exposure to HPβCD.

### 4.8. Statistics

Two-way ANOVA (analysis of variance) with Bonferroni’s multiple comparisons tests was performed using Prism 10 (GraphPad Software) to analyze DPOAE thresholds, OHC loss with age, and OHC survival under in vitro HPβCD treatment conditions. One-way ANOVA with Tukey’s multiple comparisons test was conducted using Prism (GraphPad Software) to analyze OHC loss under in vivo HPβCD treatment conditions. An unpaired two-tailed Student’s *t*-test was carried out using Prism (GraphPad Software) to analyze relative mRNA levels of *Ocm*. Values of *p* < 0.05 were considered to indicate statistical significance. To determine the required sample sizes, a priori power analyses were conducted using G*Power 3.1.9.7 [80]. For DPOAE thresholds, OHC loss with age, and OHC survival under in vitro HPβCD treatment, we selected the ANOVA: Fixed effects, special, main effects and interactions test (effect size = 0.46–0.84, alpha = 0.05, power = 0.8, number of groups = 6–8), indicating sample sizes of 3, 3, and 7 per group, respectively. For OHC loss under in vivo HPβCD treatment, we used the ANOVA: Fixed effects, omnibus, one-way test (effect size = 1.3, alpha = 0.05, power = 0.8, number of groups = 4), indicating a sample size of 3 per group. For relative mRNA levels of *Ocm*, the Means: Difference between two independent means (two groups) test was used (effect size = 3.1, alpha = 0.05, power = 0.8), resulting in a sample size of 3 per group. Effect sizes were determined based on our previous publications [37,38,40,78,81] and preliminary data.

## Data Availability

The original contributions presented in this study are included in the article/Supplementary Material. Further inquiries can be directed to the corresponding author.

## Supplementary Materials

The following supporting information can be downloaded at https://www.mdpi.com/article/10.3390/ijms27093835/s1.
