# Supplementary material for: Investigation of the Effects of Cadherin 23 and Oncomodulin on Early Progressive Hearing Loss Using a New Oncomodulin Mouse Model
Source: Int J Mol Sci. 2026 Apr 25;27(9):3835. doi: 10.3390/ijms27093835 (PMC13163798; doi:10.3390/ijms27093835)
Supplement: Supplementary file 1 [file ijms-27-03835-s001.zip › ijms-4231351-supplementary.pdf]

**Investigation of the effects of Cadherin 23 and Oncomodulin on early progressive hearing loss using a new Oncomodulin mouse model**

Mi-Jung Kim[1], Robert J. Fuentes[1], Yingjie Zhou[2], Jing Zheng[1,3]*

## Supplementary Materials

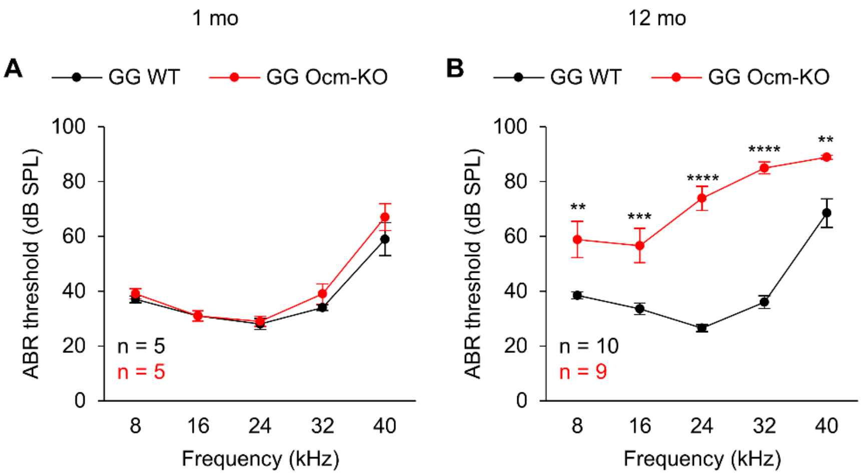


**Supplementary Figure S1**. *Ocm*$^{tm1a/tm1a}$ mice lacking a *Cdh23*$^{753G\rightarrow A}$ mutation have normal hearing at 1 month but display low to high frequency hearing loss at 12 months. (**A-B**) ABR thresholds were measured at 8, 16, 24, 32, and 40 kHz in GG WT and GG *Ocm*-KO mice at 1 (**A**) and 12 (**B**) months of age. Sample number: at 1 mo: GG WT, $n = 5$, GG *Ocm*-KO, $n = 5$; at 12 mo: GG WT, $n = 10$, GG *Ocm*-KO, $n = 9$. Data are shown as means ± SEM. Two-way ANOVA with Bonferroni's multiple comparisons tests were performed. **, $0.001 \leq p < 0.01$, ***, $0.0001 \leq p < 0.001$, ****, $p < 0.0001$. mo, month, GG WT, *Cdh23*$^{753G/753G}$;*Ocm*$^{+/+}$, GG *Ocm*-KO, *Cdh23*$^{753G/753G}$;*Ocm*$^{tm1a/tm1a}$.

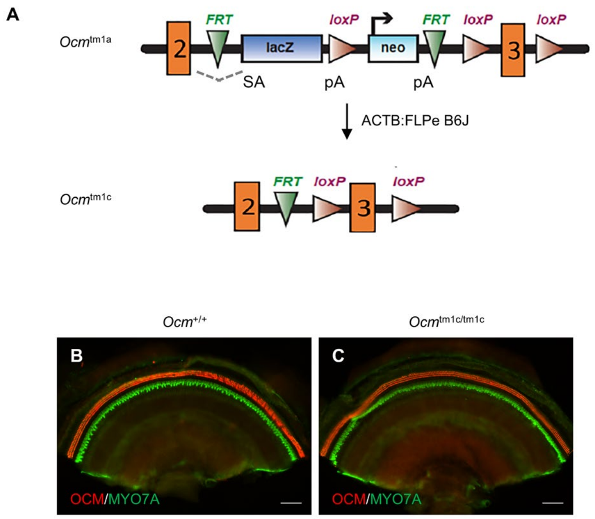


**Supplementary Figure S2**. *Ocm*$^{tm1c/tm1c}$ is a pseudo-WT mouse model. (**A**) Schematic diagram of the *Ocm*$^{tm1c}$ allele that restores *Ocm* gene expression. The *Ocm*$^{tm1c}$ strain was generated by mating the *Ocm*$^{tm1a}$ strain with ACTB:FLPe B6J mice. The Flp recombinase expressed in ACTB:FLPe B6J mice removed the *FRT*-flanked gene trapping cassette while leaving a critical exon 3 of the *Ocm* gene flanked by *loxP* sites. (**B-C**) *Ocm*$^{tm1c/tm1c}$ mice express OCM protein in the OHCs of the cochlea. OCM immunolabeling was detected in the OHCs of *Ocm*$^{tm1c/tm1c}$ mice at a similar level to WT mice. Representative immunostaining images of the cochlear whole mounts within the frequency range of 19.1-36.5 kHz from 2-month-old WT (**B**) and *Ocm*$^{tm1c/tm1c}$ (**C**) mice. Cochleae were stained for OCM (red) and MYO7A (green). Scale bar: 100 µm. WT, *Cdh23*$^{753G/753G}$;*Ocm*$^{+/+}$, *Ocm*$^{tm1c/tm1c}$, *Cdh23*$^{753G/753G}$;*Ocm*$^{tm1c/tm1c}$.

**Supplementary Movie S1.** Representative time-lapse recording of the isolated OC within the apical region of the cochlea from a 1-month-old WT mouse in the presence of 1 mM HPβCD. Isolated OC were labeled with calcein. Imaging of OC was initiated 5 minutes after exposure to HPβCD. The OC images were captured with a 1-second interval for 20 minutes. Scale bar: 20 µm. WT, *Cdh23*$^{753G/753G}$;*Ocm*$^{+/+}$.

**Supplementary Movie S2.** Representative time-lapse recording of the isolated OC within the apical region of the cochlea from a 1-month-old *Ocm*-KO mouse in the presence of 1 mM HPβCD. Isolated OC were labeled with calcein. Imaging of OC was initiated 5 minutes after exposure to HPβCD. The OC images were captured with a 1-second interval for 20 minutes. Scale bar: 20 µm. *Ocm*-KO*,* *Cdh23*$^{753G/753G}$;*Ocm*$^{tm1a/tm1a}$.
